# Targeting of MCL-1 in breast cancer-associated fibroblasts reverses their myofibroblastic phenotype and pro-invasive properties

**DOI:** 10.1038/s41419-022-05214-9

**Published:** 2022-09-14

**Authors:** Thomas L. Bonneaud, Chloé C. Lefebvre, Lisa Nocquet, Agnes Basseville, Julie Roul, Hugo Weber, Mario Campone, Philippe P. Juin, Frédérique Souazé

**Affiliations:** 1 Université de Nantes, INSERM, CNRS, CRCI2NA, 44000 Nantes, France; 2 SIRIC ILIAD, Nantes, Angers, France; 3 Omics Data Science Unit, ICO, Angers, France; 4 ICO René Gauducheau, Saint Herblain, France

**Keywords:** Cancer microenvironment, Cytoskeleton

## Abstract

Cancer-associated fibroblasts (CAF) are a major cellular component of epithelial tumors. In breast cancers in particular these stromal cells have numerous tumorigenic effects in part due to their acquisition of a myofibroblastic phenotype. Breast CAFs (bCAFs) typically express MCL-1. We show here that pharmacological inhibition or knock down of this regulator of mitochondrial integrity in primary bCAFs directly derived from human samples mitigates myofibroblastic features. This decreases expression of genes involved in actomyosin organization and contractility (associated with a cytoplasmic retention of the transcriptional regulator, yes-associated protein—YAP) and decreases bCAFs ability to promote cancer cells invasion in 3D coculture assays. Our findings underscore the usefulness of targeting MCL-1 in breast cancer ecosystems, not only to favor death of cancer cells but also to counteract the tumorigenic activation of fibroblasts with which they co-evolve. Mechanistically, pharmacological inhibition of MCL-1 with a specific BH3 mimetic promotes mitochondrial fragmentation in bCAFs. Inhibition of the mitochondrial fission activity of DRP-1, which interacts with MCL-1 upon BH3 mimetic treatment, allows the maintenance of the myofibroblastic phenotype of bCAFs.

## Introduction

In epithelial tumors, cancer-associated fibroblasts (CAFs) are an important component of a hijacked stroma and may represent up to more than half of the whole breast tumor volume. These cells are composed of different heterogeneous and plastic populations with various supporting effects on cancer cells [1, 2]. In breast cancer, different subsets of CAF have been described, differing in their expression markers, their secretions and their main characteristics (pro-tumor functions, immunoregulatory properties…) possibly linked to a different activation status. Subsets with myofibroblastic characteristics are enriched in tumor tissue and contribute to pathogenesis [3, 4]. Myofibroblastic CAFs are indeed key mediators of fibrotic tumor stroma, which is characterized by accumulation of extracellular matrix (ECM), increased tissue stiffness and breast cancer risk [5–7]. They have marked stress fibers and contract collagen, which underlies their pro-invasive properties [8, 9]. The myofibroblastic characteristics of CAFs rely on the expression of a set of genes involved in the structure of the actomyosin cytoskeleton (among them the ACTA2 encoding for the myofibroblastic activation marker α-SMA) controlled by mechano-sensitive transcription factors, YAP-TEAD and MRTF-SRF [10, 11]. These two pathways are mutually dependent and are closely regulated by the polymerization state of the actin cytoskeleton [11]. In addition to responding to mechanical stress, myofibroblast differentiation is also under the control of secreted factors [12].

Given the adverse effects of myofibroblastic CAFs on cancer progression and treatment resistance of cancer cells [13], their pharmacological manipulation and selective eradication within the tumor is therapeutically relevant [14–16]. Importantly, activated myofibroblasts are more refractory to cell death than other fibroblasts, and express higher levels of pro-survival proteins [17, 18]. Fibroblasts from patients with scleroderma rely on BCL-xL or MCL-1/BCL-xL for survival [17]. We ourselves established that MCL-1 mRNA expression positively correlates with stromal score in publicly available expression datasets from luminal breast cancers. CAFs derived from breast cancers (bCAFs), regardless of subtype, express MCL-1 at a higher level than in normal fibroblasts. This was in particular established using CAFs grown ex vivo in culture conditions that favor their myofibroblastic phenotype [13, 19]. In these cells, MCL-1 exerts its canonical function as it prevents mitochondrial outer membrane permeabilization (MOMP) in conjunction with BCL-xL [13].

In this study, we explored whether the typical expression of MCL-1 in bCAFs endowed with a myofibroblastic phenotype may constitute a vulnerability. BH3 mimetics are small molecules developed to specifically antagonize the anti-apoptotic function of various BCL-2 homologs, including MCL-1 [20]. They bind to the BH3-binding grove of anti-apoptotic proteins, which is critical for their engagement of the BH3 domain of their pro-apoptotic, MOMP-promoting, counterparts. In numerous settings, inhibition of MCL-1 grove coincidentally stabilizes MCL-1, most likely because the turn-over of this labile protein is highly dependent upon its binding to endogenous proteins harboring a BH3, or BH3-like, domain [21–23]. BH3 mimetics were initially conceived to pharmacologically favor MOMP and apoptosis in cancer cells, which are characterized by frequent alterations in the expression and activity of BCL-2 homologs. MCL-1 in particular is frequently overexpressed in many cancers, including breast cancers, and this was established to contribute to resistance to the cytotoxic effects of chemotherapy, radiotherapy and BH-3 mimetics selectively targeting other BCL-2 homologs [13, 20, 24, 25]. We also established that in luminal breast cancers, MCL-1 expression in tumor cells is extrinsically favored by paracrine effects of bCAFs. This defines MCL-1 as a target in stroma-influenced breast cancers, and advocates for a comprehensive investigation of the effects of its targeting in distinct cellular components, such as myofibroblastic CAFs, of these tumor ecosystems. We describe here that targeting MCL-1 with a BH-3 mimetic fails to induce efficient apoptosis in bCAFs. However, treatment leads to a loss of myofibroblastic features of these cells, which we ascribe to a contributing role of MCL-1 to the actomyosin dynamics of the bCAF myofibroblastic phenotype.

## Results

### MCL-1 targeting influences transcriptional programs involved in actomyosin organization/contractility in viable bCAFs

**Fig. 1:**
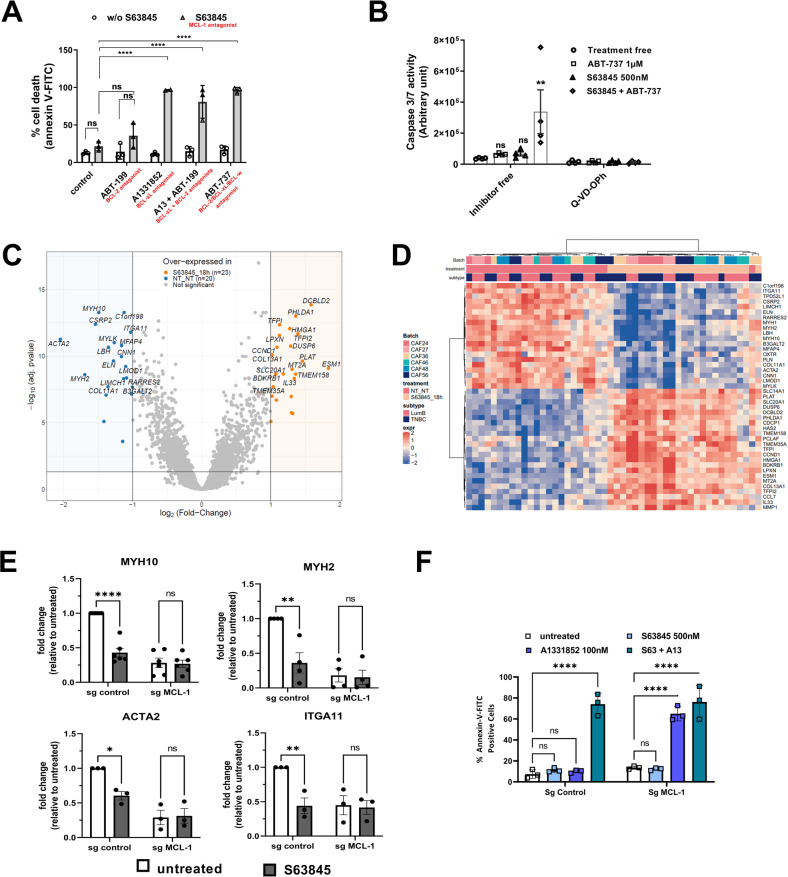
MCL-1 targeting influences transcriptional programs involved in actomyosin organization/contractility in viable bCAFs. **A** Three different primary cultures of CAFs were treated with ABT-199 (1 µM), A1331852 (100 nM), ABT-199 (1 µM) +A1331852 (100 nM) or ABT-737 (1 µM) with or without S63845 (500 nM) for 72 h in DMEM containing 1% FBS, apoptosis was measured by Annexin-V flow cytometry. Data are means ± SEM from three independent experiments. Two-way ANOVA, ****P < 0.0001, ns: not significant. **B** Caspase 3/7 activity was measured by caspase Glo assay and expressed in arbitrary unit. Data are means ± SEM from four independent experiments. Two-way ANOVA. *P < 0.05, ns: not significant **C** Volcano plot displaying differential expressed genes between S63845 treated and non-treated primary culture of CAFs from luminal B (*n* = 3) and triple negative (*n* = 3) breast cancers. The vertical axis (*y*-axis) corresponds to the mean expression value of log 10 (*q*-value), and the horizontal axis (*x*-axis) displays the log2 fold-change value. The red dots represent the upregulated expressed transcripts; the blue dots represent the transcripts whose expression is downregulated. **D** A hierarchically clustered heatmap showing the expression patterns of the 23 most extreme DE mRNA. Red and blue represent up- and downregulated expression in CAFs treated or not with S63845. Color density indicating levels of fold-change was displayed. **E** qRT-PCR of MYH10, MYH2, ACTA2 andITGA11 mRNA in CAFs Sg control or sg MCL-1 treated or not by S63845 500 nM for 18 h. Mean and SEM of three independent experiments are represented as fold-change of mRNA level related to untreated Sg control. Two-way ANOVA, *P < 0.1, **P < 0.01, ****P < 0.0001, ns: not significant. **F** CAFs sg control or silenced for MCL-1 (sg MCL-1) were treated or not with S63845 (500 nM), A1331852 (100 nM) or S63845 (500 nM) + A1331852 (100 nM) for 48 h in DMEM containing 1% FBS. Apoptosis was measured by Annexin-V flow cytometry. Data are means ± SEM from three independent experiments. *P*-value was determined by two-way ANOVA. ****P < 0.0001, ns: not significant.

We previously showed that high expression level of MCL-1 was a hallmark of activated bCAFs [13]. bCAFs systematically express the anti-apoptotic protein MCL-1 at a high level, while expression of BCL-xL is sporadic and that of BCL-2 barely detectable. To investigate whether this set of expressions corresponds to specific survival dependencies, we performed pharmacological BH-3 profiling of primary cultures of bCAFs (derived from human breast cancer samples). We treated these cells with combinations of BH-3 mimetics targeting MCL-1 (S63845), BCL-2 (ABT-199), BCL-xL (A1331852) or BCL-2/BCL-xL/BCL-w (ABT-737) and measured cell death rates (using Annexin-V binding as a readout). Results revealed that bCAFs survival did not rely on the function of a single anti-apoptotic protein (and a fortiori on that of MCL-1 alone) but rather on the combined functions of MCL-1 and BCL-xL (Fig. 1A). Accordingly, in contrast to combined targeting (using for instance S63845 + ABT-737), which triggered robust caspase 3/7 activation, targeting MCL-1 only resulted in not significant caspase-3/7 activation (Fig. 1B, S63845).

To further document the biological effects of MCL-1 targeting on bCAFs, we performed a differential gene expression (DGE) analysis on RNA-seq data from six different primary cultures of bCAF (derived from 3 triple negative and 3 luminal breast cancers) treated or not with S63845 (500 nM, 18 h). We then performed a hierarchical clustering of CAFs according to the 43 differentially expressed genes, and plotted them accordingly in a heatmap (Fig. 1C, D). As expected, CAFs were split into two clusters according to the treatment received. No additional clustering was observed according to cancer subtype origin (TNBC or Luminal). We confirmed by RT-PCR the S63845-induced significant down-regulation of a selected set of genes (MYH10, MYH2, ACTA2 and ITGA11) compared to untreated CAFs (Fig. 1E, sg control). To confirm that MCL-1 contributes to the expression of this set of genes, we knocked down MCL-1 expression in primary bCAFs by CRISPR-CAS9-based genome editing. Genetic deletion of MCL-1 in bCAFs phenocopied the transcriptomic effects of S63845 treatment. Moreover, S63845 has no additional effect on gene expression in MCL-1 deficient CAFs (Fig. 1E, sgMCL-1). MCL-1 knock down was validated by the significant decrease in MCL-1 expression and loss of MCL-1 stabilization induced by S63845 (Supplementary Fig. 2D). The functionality of MCL-1 loss was assessed by the ability of the BCL-xL antagonist (A1331852) to trigger cell death in KD MCL-1 whereas it did not in control sg CAFs (Fig. 1F, Bottom).

### Myofibroblastic features of bCAFs are MCL-1 dependent

**Fig. 2:**
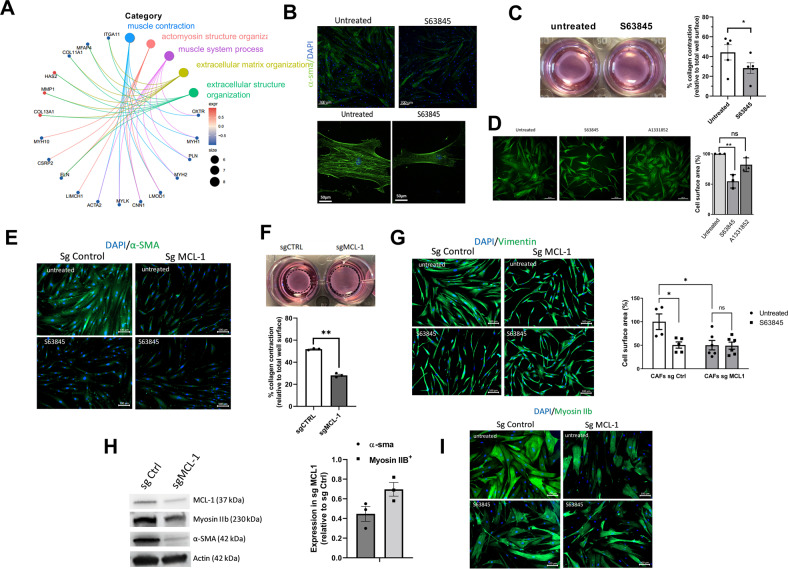
MCL-1 antagonist mitigates myofibroblastic features. **A** Gene Ontology (GO) pathway enrichment analysis based on DGE results. **B** Representative immunofluorescence of α-smooth muscle actin (α-SMA) (green) in CAF treated or not with S63845 for 18 h, nuclei were stained in blue (4’,6-diamidino-2-phenylindole, DAPI) (*n* = 3). **C** Representative images of CAFs contraction of collagen gels. Quantification of collagen gel contraction by CAFs treated or not with S63845. 100% represents the total surface area of the well (*n* = 5) Student *t*-test, *P < 0.05. **D** Representative immunocytochemistry of Vimentin immunostaining used to determine cells surface area of 3 different CAFs treated or not with S63845 500 nM or A1331852 100 nM for 18 h. Cells area were determined with imageJ software by measuring average of cell surface area from 200 cells for each condition, results are expressed in arbitrary unit relative to untreated cells. Anova, **P < 0.01, ns: not significant. **E** Representative immunofluorescence of α-smooth muscle actin (α-SMA) (green) in CAFs knock down for MCL-1 (Sg MCL-1) or not (Sg control), nuclei were stained in blue (4’,6-diamidino-2-phenylindole, DAPI) (*n* = 3). **F** Representative images of CAFs contraction of collagen gels. Quantification of collagen gel contraction by CAFs knock down for MCL-1 (Sg MCL-1) or not (Sg Control). 100% represents the total surface area of the well. (*n* = 3) Student *t*-test, **P < 0.01. **G** Representative immunocytochemistry of Vimentin immunostaining used to determine cells surface area of 3 different CAFs Sg Control or Sg MCL-1. Cells area were determined with imageJ software by measuring average of cell surface area from 200 cells for each condition, results are expressed in arbitrary unit relative to untreated cells. Anova, *P < 0.05, ns: not significant. **H** (Left) Representative experiment of protein expression level (MCL-1, Myosin IIB, α-SMA) evaluated using western blots, Actin expression was used as loading control. (Right) Quantification of the amounts of α-SMA or Myosin IIB protein band relative to Actin in CAFs Sg MCL-1, results are expressed as a ratio relative to Sg Control. **I** Representative immunofluorescence of Myosin IIB (green) in CAFs knock down for MCL-1 (Sg MCL-1) or not (Sg control), nuclei were stained in blue (4’,6-diamidino-2-phenylindole, DAPI) (*n* = 3).

Gene Ontology (GO) pathway enrichment analysis based on DGE results indicated that pharmacological inhibition of MCL-1 in bCAFs was mainly associated with a decreased expression of genes involved in extracellular matrix and actomyosin structure organizations and in contractility (Fig. 2A). Among these genes, the majority is known to be involved in myofibroblastic differentiation and are bona fide CAF markers such as ACTA2 (coding for α-SMA), MYH10 (coding for myosin IIB), COL11A1 or ITGA11 (Figs. 1C, E, and 2A). To validate the impact of MCL-1 targeting on bCAFs myofibroblastic phenotype, we evaluated the effects of S63845 treatment and of MCL-1 knock down on α-SMA and myosin IIB protein expressions, CAF contractile force and CAF surface area. Either manipulation induced a decrease in α-SMA and myosin IIB expressions (Fig. 2B, E, H, and I), inhibited collagen contraction ability (Fig. 2C, F) and reduced CAFs surface area (Fig. 2D, G).

### Targeting of bCAFs-expressed MCL-1 decreases invasiveness and pro-invasive effects on cancer cells without affecting their proliferation

**Fig. 3:**
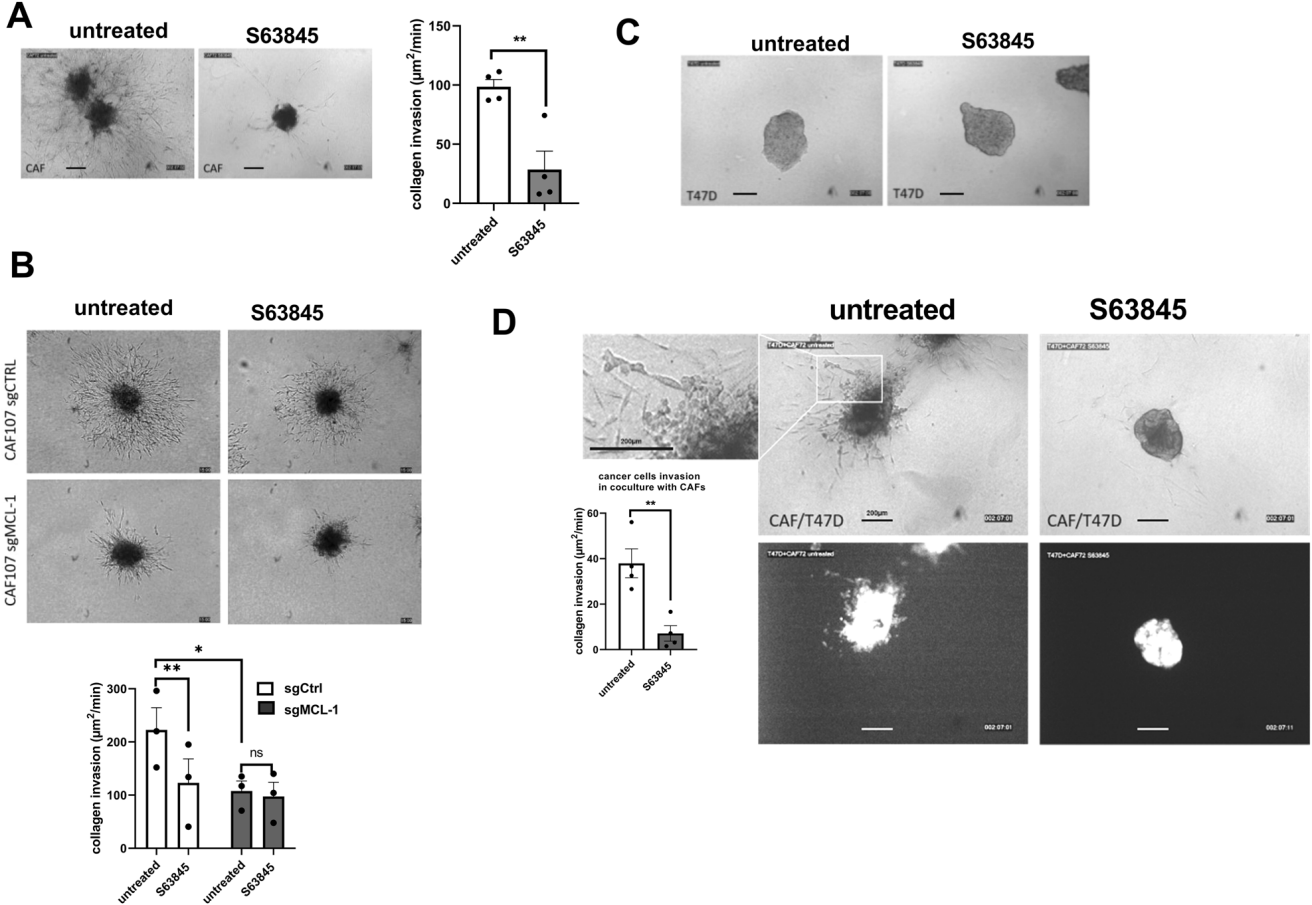
MCL-1 targeting in bCAFs decreases invasiveness and pro-invasive effects on cancer cells. **A** CAFs treated or not with S63845 **B** CAFs sg control or sg MCL-1 treated or not with S63845 **C** T47D cancer cells or **D** multicellular CAF + T47D-eGFP 3D spheroid invasion into collagen in presence or not of S63845 (500 nM) was monitored up to 48 h using microscopy time-lapse. Representative images of tumor cell invasion are shown. Quantification (3 spheroids per CAF in 3 or 4 different CAFs) are expressed in invaded surfaces (including cells number and distance) expressed in µm^2^/units of time (min).

We investigated the consequence of S63845-induced phenotypic changes on the invasive properties of bCAFs. To this end, we analyzed the influence of MCL-1 expression on invasion of bCAFs spheroid in type I collagen matrix. As shown in Fig. 3A, B, whereas bCAFs rapidly invaded the collagen matrix in these 3D assays, MCL-1 targeting (by S63845 in Fig. 3A or genetic silencing in Fig. 3B) significantly reduced bCAFs invasion. As bCAFs were described to promote cancer cells invasion [8], we also investigated fibroblast-led invasion of breast cancer cells and the influence of S63845 treatment in 3D coculture assays. Cancer cells were engineered to express GFP as a discriminating marker and we chose to use luminal T47D breast cancer cells that, under our experimental conditions, did not invade the collagen matrix when grown as spheroids in the absence of bCAFs (Fig. 3C). Upon coculture with bCAFs as 3D-multicellular tumor spheroids in type I collagen, GFP-positive T47D cancer cells invaded collagen and this was blocked by S63845 treatment (Fig. 3D) without inducing cancer cell death (Supplementary Fig. 1A). Loss of myofibroblastic features and of contractility noted upon MCL-1 neutralization are accompanied by a decrease in the pro-invasive properties of bCAFs.

**Fig. 4:**
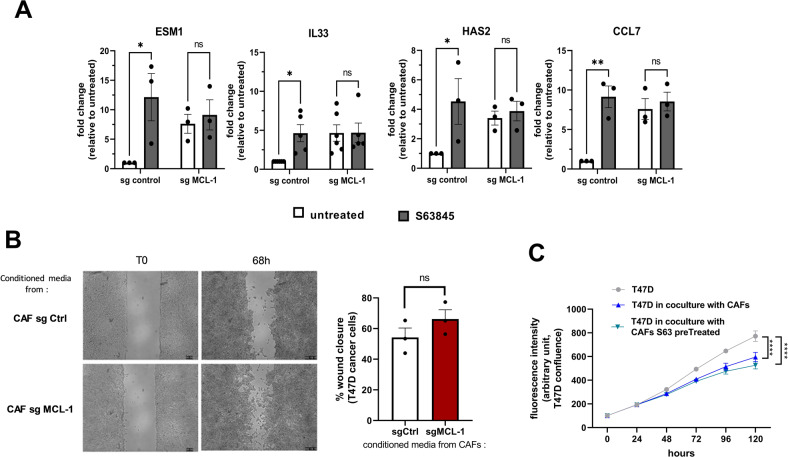
MCL-1 targeting in bCAFs does not induce cancer cell migration or proliferation. **A** qRT-PCR of ESM1, IL33, HAS2 and CCL7 mRNA in CAFs Sg control or sg MCL-1 treated or not by S63845 500 nM for 18 h. Mean and SEM of three to five independent experiments are represented as fold-change of mRNA level related to untreated Sg control. Two-way ANOVA, *P < 0.1, **P < 0.01, ns: not significant. **B** (Left) Representative images from in vitro scratch wound-healing assays of T47D cancer cells in presence of conditioned media from CAFs Sg control or CAFs Sg MCL-1 at T0 and after 68 h. (Right) Quantification of wound closure expressed in percentage of total wound recovery at indicated time points during the scratch wound assay. Student *t*-test (ns: not significant). **C** Proliferation of T47D-eGFP cancer cells in coculture with CAFs pretreated or not with S63845 measured. Confluency was measured using time-lapse video-microscopy (incucyte live cells analysis) during 120 h. Student *t-*test (****P < 0.0001).

Our DGE analysis revealed that in addition to the down-regulation of genes implicated in myofibroblastic features, S63845 induced the transcription of some inflammatory related genes such as IL33, CCL7, ESM1, Has2, and TMEMs (Fig. 1C, D and Fig. 4A). As shown in Fig. 4A, the same effect was observed on mRNA expression in CAFs knocked down for MCL-1. These inflammatory factors may influence cancer cell migration and proliferation [26–28]. However, neither the silencing of MCL-1 in CAFs nor its inhibition by S63845 detectably increased cancer cell migration or proliferation, as evaluated by wound-healing assays and time-lapse video-microscopy (Fig. 4B, C). It should be noted that under the conditions of coculture used here, the decrease in cancer cell proliferation observed when CAFs were present may be caused by metabolic competition between the two cell types or by the technique used.

### The phenotypic effects on bCAFs of MCL-1 targeting rely on mitochondrial fragmentation

**Fig. 5:**
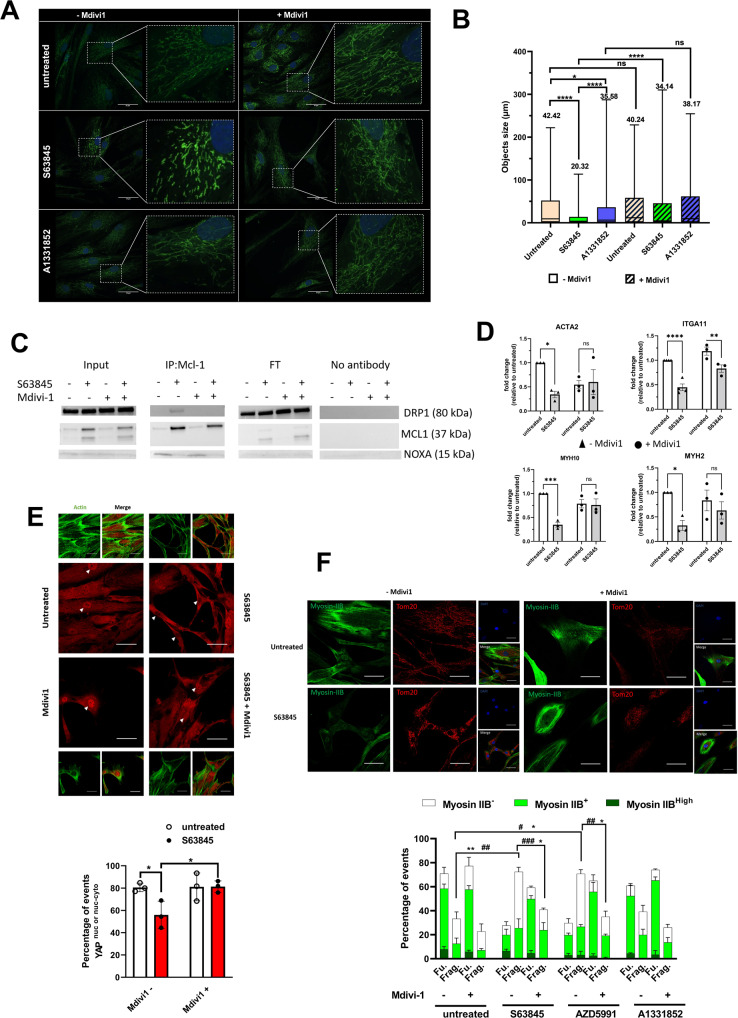
MCL-1 targeting induces changes in actomyosin structure organization in a DRP-1-dependent manner. **A** Mitochondrial fission was detected by TOM20 staining in CAFs treated or not with S63845 500 nM or A1331852 100 nM for 18 h in the presence or not of Mdivi-1 50 µM. Scale bar = 50 μm. **B** Quantification of mitochondrial network length was performed by measuring the size of TOM-20 labeled structures on deconvolved images in 10 cells/condition in 3 independent experiments. Objects smaller than 0.5 µm were not considered. **C** Representative co-immunoprecipitation of MCL-1 and DRP-1 and western blotting for MCL-1, DRP1, or NOXA in CAFs treated or not with S63845 in presence or not of Mdivi-1 (*n* = 3). FT: Flow Through. **D** qRT-PCR of MYH10, MYH2, ACTA2, and ITGA11 mRNA in CAFs treated or not by S63845 500 nM for 18 h in the presence or not of Mdivi-1. Mean and SEM of three independent experiments are represented as fold-change of mRNA level related to untreated control. Two-way ANOVA, *P < 0.1, **P < 0.01, ***P < 0.001, ****P < 0.0001, ns: not significant. **E** Quantification (percentage of cells positive for nuclear YAP or nuclear and cytosolic YAP) (Bottom) and confocal image (Top) of YAP staining (red) and FITC-phalloidin (green) in CAFs treated or not with S63845. Randomly generated images set names were randomized for analysis. Around sixty to one-hundred cells were analyzed per condition. Counting events were done manually through NIS-Elements software (Nikon software). Fluorescence intensity and positivity staining were determined by positive and negative control comparison, dependent on the experiment. The nuclei were counterstained with DAPI (blue). Scale bar = 50 μm. Two-way ANOVA, *P < 0.05. **F** Quantification (Bottom) and confocal image (Top) of Myosin IIB staining (green) and immunofluorescence of TOM-20 (red) in CAFs treated or not with different BH3 mimetics (as indicated) in the presence or not of Mdivi-1. Randomly generated images set names were randomized for analysis. Around sixty to one-hundred cells were analyzed per condition. Counting events were done manually through NIS-Elements software (Nikon software). Fluorescence intensity and positivity staining were determined by positive and negative control comparison, dependent on the experiment. The nuclei were counterstained with DAPI (blue). Scale bar = 50 μm, Two-way ANOVA, ^###^*P* < 0.001, ^##^*P* < 0.01, ^#^*P* < 0.05 for myosin IIB-, **P < 0.01; *P < 0.05 for fragmented mitochondria.

We finally explored the mechanistic basis of the effects of MCL-1 inhibition on bCAFs. In addition to MOMP, MCL-1 was shown to regulate the dynamics between mitochondrial fusion and fission in human pluripotent stem cells (hPSCs) and in hPSCs-derived cardiomyocytes [29], and S63845 treatment of cardiomyocytes was shown to promote mitochondrial fission [30]. A more thorough investigation of the impact of S63845 treatment on the mitochondrial network of bCAFs showed that it reduced mitochondrial length in bCAFs whereas BCL-xL antagonist (with A1331852) did not (Fig. 5A, B). S63845-induced mitochondrial ultrastructural defects did not coincide with detectable changes in oxidative phosphorylation (measured with a Seahorse, Supplementary Fig. 1B) and mitochondrial mass (evaluated by total TOM-20 expression, Supplementary Fig. 1C). The effect of S63845 was prevented by co-treatment with Mdivi-1, an inhibitor of dynamin-related protein 1 (DRP-1) impairing Drp1 oligomerization, its self-assembly in rings in association with mitochondria and its subsequent GTPase activity, suggesting that it relies on activation of the endogenous mitochondrial fission machinery of which this protein is a critical member (Fig. 5A). Mechanistically, and in line with previous reports in numerous other cell types, bCAFs treatment with S63845, but not with A1331852, enhanced MCL-1 protein levels (Supplementary Fig. 1D, E). MCL-1 accumulated preferentially at the mitochondria as illustrated by the colocalization of MCL-1 and TOM20 seen in immunocytochemistry (Supplementary Fig. 1E). S63845 treatment enhanced interactions between MCL-1 and DRP1, as judged in co-immunoprecipitation assays, in a Mdivi-1-sensitive manner. In contrast, S63845 prevented interactions between MCL-1 and NOXA (Fig. 5C).

We also showed that the S63845-induced down-regulation of a set of genes (MYH10, MYH2, ACTA2 and ITGA11) is impaired in presence of Mdivi-1 for most tested genes (Fig. 5D). It is to note that this Mdivi-1-sensitive effect on gene regulation is not found on the upregulated genes (Supplementary Fig. 2A).

Transcription factor “activation” analysis based on S63845-induced differential gene expression hinted on SRF as a candidate transcription factor possibly involved in the observed down regulations (Supplementary Fig. 2B). However, we could not detect any change in the nuclear localization of SRF or of its cofactor, MRTF, upon treatment (data not shown). SRF/MRTF-regulated genes were shown to co-depend on YAP in bCAFs [11]. Using confocal immunofluorescence, we found that the proportion of bCAFs exhibiting nuclear localization of YAP (including nuclear and cytosolic) was decreased upon pharmacological inhibition (Fig. 5E, Top and Bottom). Without ruling out the involvement of additional transcriptional factors, this argues that cytoplasmic retention of YAP may contribute, at least in part, to the transcriptional effects of MCL-1 targeting. The effects of MCL-1 targeting on MYH10, MYH2, ACTA2 and ITGA11 mRNA down-regulation (Fig. 5D) and on YAP cytoplasmic retention (Fig. 5E) were reversed by Mdivi-1 treatment. To further document the link between MCL-1 regulated mitochondrial dynamics and myofibroblastic features, we co-evaluated myosin-IIB (MYH10) expression since this protein has an established role in CAFs invasion ability [10, 31] and since its mRNA expression was found downregulated by S63845 and mitochondrial networks (distinguished between fragmented and fused by immunolabelling of the outer mitochondrial membrane protein TOM20 Fig. 5F; individual values are shown in Supplementary Fig. 3) in bCAFs cultured in the absence or presence of S63845. S63845-induced mitochondrial fragmentation was accompanied by a decrease in Myosin-IIB expression in bCAFs with 60% of CAFs not expressing it (mostly exhibiting fragmented mitochondria) versus 30% in the control (Fig. 5F, Top and Bottom). Mdivi-1 co-treatment prevented these effects (Fig. 5F). This was recapitulated by treatment with another MCL-1 targeting BH3 mimetic (AZD5991) but not by a BCL-xL targeting BH3 mimetic (A1331852).

These results suggest that acute pharmacological inhibition of MCL-1 contributes to a loss of the myofibroblastic phenotype of bCAFs by promoting mitochondrial dynamic imbalance. Of note, we failed to detect steady state mitochondrial fragmentation in MCL-1-deficient CAFs (data not shown), and Mdivi-1 treatment did not restore the long-term effects of MCL-1 knock down on the expression of “myofibroblastic genes” (Supplementary Fig. 2B). However, the effect of S63845 on mitochondrial fragmentation was lost in MCL-1-deficient CAFs but not in BAX-BAK-deficient CAFs (obtained by CRISPR-CAS9) (Supplementary Fig. 2D). Acute MCL-1 inhibition and long-term MCL-1 deletion may thus mitigate the myofibroblastic features of bCAFs by distinct mechanisms, underscoring the complexity of MCL-1 contribution to bCAFs phenotypes.

## Discussion

We herein present a novel and unexpected role for MCL-1: its contribution to the myofibroblastic phenotype and pro-invasive capacity of cancer-associated fibroblasts. Numerous studies showed that tumor intrinsic deregulation of MCL-1 expression/activity promotes breast cancer progression and treatment resistance. We also previously showed that in luminal breast cancers, MCL-1 expression in tumor cells, leading to a survival advantage, was extrinsically favored by neighboring CAFs, which themselves exhibit high MCL-1 expression [13]. Our study thus brings support to the notion that MCL-1 targeting may improve the treatment of breast cancer [20, 25] while putting forth an effect not only on cancer cells but also on non-malignant cells that interact with them. One implication is that the therapeutic potential of BH3 mimetics inhibiting MCL-1 may be preclinically overlooked if reductive models using only tumor cells are used. Our study highlights instead that diverse cellular components of the microenvironment may experience an intense pressure on BCL-2 family dependent mechanisms during tumor growth. This advocates for the use of assays that include cellular components of the microenvironment to investigate the effects of targeted agents such as BH3 mimetics, but also of cytotoxic compounds that indirectly impact on the BCL-2 network.

While in some models BH3 mimetics antagonizing MCL-1 efficiently counter tumor progression [32], the sole targeting of MCL-1 in solid tumors proved little efficiency to trigger cell death on its own, both in xenograft and PDX immunodeficient mice models [25, 33]. Likewise, we observed that treatment of bCAFs with S63845 only induced moderate apoptotic rates. Moreover, treatment with a BCL-xL antagonist (A1331852), which alone did not induce bCAF death, did so in combination with S63845. Inefficient killing of the latter as a single agent may thus ensue from dynamic sequestration by BCL-xL of pro-apoptotic proteins released from MCL-1 upon treatment. We cannot rule out, however, that S63845 inefficiency when used alone may stem from the lack of binding of pro-apoptotic proteins to MCL-1 at baseline, (leaving “empty” MCL-1 available to engage pro-apoptotic proteins released from BCL-xL by A1331852, and to protect from cell death). In all cases, our functional studies show that MCL-1 combines its anti-apoptotic activity with that of BCL-xL to maintain bCAFs survival. Consistently, CRISPR-Cas9 engineered knock down of MCL-1 in bCAFs dramatically sensitized them to a BCL-xL inhibitor.

Even though the pro-apoptotic effects of MCL-1 targeting are restrained by BCL-xL activity, MCL-1 targeting by itself modifies the tumorogenic phenotype of bCAFs by mitigating their myofibroblastic features and invasive properties. Our study evokes reports indicating that MCL-1 is not only an anti-apoptotic protein determining life death decisions but also a more subtle regulator of the phenotype, and the mechanical properties in particular, of various cell types. A role for MCL-1 in cellular differentiation was defined in cardiomyocytes, and stem cells [29, 30] and the antagonistic effects of its pharmacological inhibition by S63845 in cardiomyocyte function and contractility was described [30]. This latter effect, which corroborates our study, may be the basis for the dose limiting cardiotoxic effect of MCL-1 antagonists when used in the clinic [34]. The therapeutic window for exploiting the effects of MCL-1 targeting on a myofibroblastic tumor stroma described here may thus be narrow, unless specific tumor delivery strategies intervene.

Our study indicates a strong dependency on MCL-1 of the myCAF phenotype. CAFs are an heterogenous component of the cellular microenvironment as underlined by numerous studies identifying phenotypically distinct CAFs subsets in various cancers [3, 19, 35–38]. There seems to be some consensus regarding the existence of perivascular CAFs and of ECM rich CAFs. The latter population is enriched by our isolation protocol [13], and heterogenous in itself. Breast ECM-CAF encompass, in molecular subtype dependent and patient specific proportions, CAF subsets endowed with an inflammatory phenotype (iCAFs) and other myofibroblastic CAFs (myCAFs). A similar dichotomy was described in pancreatic ductal adenocarcinoma models and clinical samples, where IL1-driven iCAFs and TGFb driven myCAFs were described [39]. The latter study unravels an “activation” process with a transition from normal fibroblasts to a single CAF phenotype, later diverging to either iCAF or my CAF. MyCAFs are of particular relevance as they exert an immunosuppressive effect and are associated with primary resistance with immunotherapy. Further studies are required to determine at which step(s) of this CAF “activation” MCL-1 is involved. In particular, understanding whether MCl-1 expression and interactome are differently regulated in distinct subsets during activation is of particular interest.

One of the most exciting implication from our study is that it shows that CAFs “activation”, or at least some steps of this complex and tumorogenic process may be reversible, as myofibroblastic features can be pharmacologically reversed using a small molecule targeting MCL-1. Our data argue that the molecular basis for the effects of S63845 in bCAFs are not canonical, in that they seem to associate neither to the release of one or more pro-apoptotic proteins from MCL-1 nor to obvious MOMP. Consistently, the effects of S63845 do not necessitate BAX-BAK expression: MCL-1 targeting induced mitochondrial fragmentation BAX-BAK KD bCAFs (Supplementary Fig. 2D), note that these cells are resistant to S63845 combined with A1331852 (Supplementary Fig. 2E). We instead ascribed the effects of S63845 to induction of mitochondrial fragmentation, as changes induced in the actomyosin network by MCL-1 targeting where counteracted by inhibition of DRP-1. This evokes reports of an active, possibly groove independent role for MCL-1 in mitochondrial fission in mouse embryonic fibroblasts [40] and of the effects of S63845 treatment on mitochondrial fission and on the inhibition of cardiomyocyte function and contractility [30]. MCL-1 is a very labile protein with a half-life protein around 30 min [13, 24] stabilized in many cell types by occupancy of its BH3-binding groove by endogenous proteins or S63845 [21–23, 34, 41]. This also occurs in bCAFs as S63845 induces an increase in MCL-1 protein expression level and further studies are required to identify bCAF expressed proteins involved in MCL-1 turn-over. Stabilization of MCL-1 in S63845 treated bCAFs coincides with its interaction with DRP-1 in a Mdivi-1-sensitive manner. MCL-1 was already described to interact with DRP-1 (in addition to OPA1), for instance in human pluripotent stem cells (hPSCs) and in hPSC-derived cardiomyocytes [40, 42] [29, 30]. The most likely explanation for the S63845 short-term effect in bCAFs is that the treatment allows groove independent interaction of MCL-1 with DRP-1 at mitochondria (where MCL-1 was found to accumulate), leading to organelle fission and subsequent loss of myofibroblastic features. MCL-1 also appears to contribute to the myofibroblastic features of CAFs by other mechanisms, unrelated to acute mitochondrial fission, as MCL-1 knock down CAFs are phenotypically altered in a Mdivi-1 manner without exhibiting overt mitochondrial fragmentation. Further studies are required to unravel how loss of MCL-1 impacts on myofibroblastic features in the long-term. As inflammatory and myofibroblastic CAFs phenotypes are mutually exclusive, one may speculate that MCL-1 loss (which induces the expression of inflammatory genes in a Mdivi-1 independent manner) may erode the latter by favoring the former.

Our description of the effects of S63845 unravels a link, in bCAFs, between mitochondrial fusion/fission dynamics, actomyosin cytoskeleton and the expression of a set of myofibroblastic genes. These are known to be regulated by transcription factors such as SRF-MRTF and YAP-TEAD whose mechano-dependent activity is itself dependent on an active actomyosin network [11]. We propose that S63845 treatment interferes with this positive feedback loop, as it prevented the nuclear localization of YAP in the same time as it prevented actomyosin fibers formation. Numerous studies have established an influence of mitochondrial function on cytoskeleton organization and/or mechano-dependent transcription factors [43, 44]. It should be noted that we observed changes in myofibroblastic features during S63845 treatment whereas we could not detect any change in mitochondrial mass or oxidative phosphorylation. We thus propose that the biological effect we describe relies on a more direct link between the mitochondrial fusion-fission machinery and the cytoskeleton.

The effects of S63845 (and of the related AZD molecule) and of MCL-1 knock down herein reported pleads for the use of MCL-1 inhibitors for the treatments of cancers with stromal evolutions into myofibroblasts (with advantages and limitations discussed above) but also give indications about the influence of current treatments might have on the tumor stroma. It is relevant here to note that MCL-1 mRNA and protein expression levels are inhibited by anthracyclines, which are part of the chemotherapeutic cocktail used for breast cancer therapy. Analyzing the clinical effects of chemotherapy on CAF expressed MCL-1 may thus help predict tumor response.

## Methods and Materials

### Cell culture and reagents

Fresh human mammary samples were obtained from treatment naive patients with invasive carcinoma after surgical resection at the Institut de Cancérologie de l’Ouest, Nantes/Angers, France. As required by the French Committee for the Protection of Human Subjects, informed consent was obtained from enrolled patients and protocol was approved by Ministère de la Recherche (agreement no.: DC-2012-1598) and by local ethic committee (agreement no.: CB 2012/06). Breast cancer-associated fibroblasts (bCAFs) isolation and characterization were previously described in Louault et al. [13].

For the CRISPR Cas9-induced knock-out (KO) primary bCAFs, single-guide (sg) RNA sequences targeting human genes were designed using the CRISPR design tool (http://crispor.tefor.net). The guide sequences CGCGGTGACGTCGGGGACCT were cloned in the plentiCRISPRV2 vector that was a gift from Feng Zhang (Addgene plasmid # 52961) [45]. Cells were selected using 1 μg/ml puromycin and protein extinction were confirmed by immunoblot analysis.

The human breast cancer cell lines T47D were purchased from American Type Culture Collection (Bethesda, MD, USA), GFP was introduced by lentiviral infection with PFG12 (insert EGFP) from Addgene. FG12 was a gift from David Baltimore (Addgene plasmid # 14884; http://n2t.net/addgene:14884; RRID:Addgene_14884).

BH3 mimetics, S63845 and ABT-199 (Chemieteck, Indianapolis, IN, USA), AZD-5991 and A1331852 (MedChemExpress, NJ, USA), ABT-737 (Selleckchem, Houston, TX, USA), and Q-VD-OPh (Sigma-Aldrich, Saint-Louis, MO,USA) and Mdivi-1 (Sigma-Aldrich, Saint-Louis, MO, USA) were diluted in DMSO and used at the indicated concentrations.

### Apoptosis assays

The activity of caspase-3/7 was measured by Caspase-Glo-3/7 assay kit according to the manufacturer’s instructions (Promega). Cell death was assessed by an Annexin-V FITC-binding assay (Miltenyi, France) performed according to manufacturer’s instructions. Flow-cytometry analysis was performed on an Accuri C6 flow cytometer (BD biosciences San Jose, CA, USA).

### Immunocytochemistry

Cells were fixed in PBS containing 4% paraformaldehyde/4% sucrose for 15 min. Cells were permeabilized for 5 min at room temperature in 0.25% Triton-X-100 in PBS, washed twice with PBS, and incubated for 30 min at 37 °C in PBS containing 10% BSA. Cells were incubated overnight at 4 °C with primary antibodies diluted in PBS containing 3% BSA. Antibodies used were as follows: mouse anti alpha-SMA (1:200, Invitrogen, Carlsbad, CA, USA); Myosin-IIB (1:1000, BioLegend, San Diego, CA, USA); YAP1 (1:50, Proteintech, Manchester, UK); TOM20 (1:80, Santa Cruz Biotech., Dallas, TX, USA); MCL-1 (1:800, Cell Signaling Technology, Danvers, MA, USA); Vimentin (1:100, Merck, Darmstadt, Germany). Actin labeling were performed with Alexa 488-conjugated Phalloidin (1:100, Cell Signaling Technology, Danvers, MA, USA). After washing, cells were incubated for 90 min at room temperature with the appropriate Alexa 488-conjugated secondary antibodies diluted in PBS containing 3% BSA. Cells were washed with PBS and mounted with ProLong Diamond Antifade Reagent with DAPI (Invitrogen, Carlsbad, CA, USA). Fluorescence images were acquired with Nikon A1 Rsi Inverted Confocal Microscope (Nikon, Tokyo, Japan) with NIS-Elements software (Nikon). Images set position were generated randomly through NIS-Elements JOBS module (Nikon).

### Immunoblot analysis

Cells were re-suspended in lysis buffer (1% SDS; 10 mM EDTA; 50 mM Tris-Hcl pH 8.1; 1 mM PMSF; 10 μg/ml aprotinin; 10 μg/ml leupeptin; 10 μg/ml pepstatin; 1 mM Na3VO4 and 50 mM NaF). For western blotting, following SDS–PAGE, proteins were transferred to 0.45 µM nitrocellulose membranes using Trans-Blot® Turbo™ Transfer System Cell system (Bio-Rad). The membrane was then blocked in 5% nonfat dry milk TBS 0.05% Tween 20 and incubated with primary antibody overnight at 4 °C. Blots were incubated with the appropriate secondary antibodies for 1 h at room temperature and visualized using the Chemi-Doc XRS + system (Bio-Rad). The used primary antibodies were anti-α-SMA (Invitrogen) (MA5 11547), anti-Myosin-IIB (BioLegend) (909901), anti-MCL-1 (Santa Cruz) (sc-819) anti-Bax (Dako) (A3533), anti-Bak (Cell signaling) (3814), anti-Tom20 (ab186734) (Abcam) anti-β-ACTIN (Millipore) (MAB1501R). Whole uncropped images of the original western blots are presented in Supplementary Fig. 4.

### Co-Immunoprecipitation

CAFs were collected in lysis buffer (CHAPS 1%, TRIS-HCl 20 mM pH=7.5, NaCl 150 mM, EDTA 1 mM pH = 8.0, proteases and phosphatases inhibitors). Co-Immunoprecipitation was performed using PureProteome™ Protein A Magnetic Beads (Millipore, LSKMAGA10). Protein extracts were first precleared using 10 µl of beads for 200 µg of proteins (1-h incubation at 4 °C). 2 µl of anti-MCL-1 antibody (Cell signaling mAb #94296) and 10 µl of beads were then used for 200 µg of proteins following manufacturer’s instructions. For western blotting, DRP-1 antibody (MA5-26255, Thermo Fisher Scientific, Waltham, MA, USA) was used as primary antibody (1:1000) and the same anti-MCL-1 primary antibody (1:1000). Clean-Blot™ IP Detection Reagent (HRP) (Thermo Scientific #21230) was used as secondary antibody (1:1000). Whole uncropped images of the original western blots are presented in Supplementary Fig. 4.

### Seahorse

OCR measurements were performed using the Seahorse XF HS Mini Analyzer (Seahorse Bioscience). Cells were plated in duplicate in 8-well seahorse plate (7000 cells per well) and treated with S63845 500 nM for 18 h. Then, medium was replaced by glutamine/glucose/pyruvate-free DMEM (no sodium bicarbonate) adjusted to pH 7.4 and incubated for 45 min at 37% in a CO_2_-free incubator. OCR was normalized to cell number ratio determined by cell counting at the end of the experiment.

### Analysis of mRNA expression data in CAFs

Sample were analyzed according to the following protocol [46]. Gene expression profiles are generated by parsing the alignment files (.bam) and counting for each sample the number of UMIs associated with each gene. Reads aligned on multiple genes, containing more than three mismatches with the reference sequence or having a polyA pattern are discarded. Finally, a matrix containing the counts of all genes on all samples is produced. The expression values, corresponding to the absolute abundance of mRNAs in all samples, is then ready for further gene expression analysis. From normalized count matrix (DEseqd2 package) [47], batch effect was corrected according to CAF patient origin using Limma package. Differentially expressed genes were calculated using ebayes function (Limma) and filtered in with an adjusted p-value inferior to 0.05 and a log2 fold-change superior to 1. Heatmap was built with complexHeatmap package using euclidean distance and ward D2 clustering method. Pathway enrichment analysis was done using clusterprofiler package.

Transcription factor activation was determined using gene set enrichment analysis (GSEA, fgsea package) using “TFT_Legacy” from molecular signature database (msigdbr package) and *t*-test-ranked genes.

### Collagen gel contraction assay

Fibroblasts (100,000 cells) were re-suspended in 500 μl collagen type I suspension (1 mg/ml) (BD/corning) (354249) in DMEM supplemented with 1% FBS. Cell suspension was cast into each well of 24-well tissue culture plate and incubated at 37 °C for 1 h in order to facilitate gelation. Following this, gels were released from the surface of the culture well using a sterile tip. Contraction between the different conditions was evaluated at 3 h.

### RNA isolation and quantitative real-time PCR

Total RNA was isolated using Nucleospin RNA (Macherey-nagel, Hoerdt, France) and transcribed into cDNA by Maxima First Strand cDNA synthesis Kit (Thermo scientific). Quantitative RT-PCR (qPCR) was performed using the EurobioGreen qPCR Mix Lo-Rox with qTOWER (Analityk-jena, jena, Germany). Reaction was done in 10 μl final with 4 ng RNA equivalent of cDNA and 150 nM primers. Relative quantity of mRNA was estimated by Pfaffl method [48] and normalized on the average relative quantity of two housekeeping genes.

RPLP05’-AACCCAGCTCTGGAGAAACT/CCCCTGGAGATTTTAGTGGT-3’

GAPDH5’-CAAAAGGGTCATCATCTCTGC/AGTTGTCATGGATGACCTTGG-3’

ACTA25’-CTATGCCTCTGGACGCACAACT/CAGATCCAGACGCATGATGGCA-3’

MYH25’-GTCTGCCAACTTCCAGAAGC/CAGCTTGTTCAAATTCTCTCTGAA-3’

MYH105’-GACTGAGGCGCTGGATCTGT/AAAGCAATTGCCTCTTCAGCC-3’

ITGA115’-CGGCCTCCAGTATTTTGGCT/GGAGGCTGGCATTGATCTGA-3’

ESM1 5’-AAAGGCTGCTGATGTAGT/TCTCTGAGGTGGCATACG-3’

IL33 5’-AATCAGGTGACGGTGTTG/ACACTCCAGGATCAGTCTTG-3’

CCL7 5’-ACCACCAGTAGCCACTGTCC/GAGGAGCATCCCACAGTTTT-3’

HAS2 5’-TCGCAACACGTAACGCAA/ACTTCTCTTTTTCCACCCCATTT-3’

### 3D spheroid collagen invasion

3D-spheroids were obtained by re-suspending cells at a concentration of 1.25 × 10^4^ cells/ml in 20% Methyl-cellulose/80% DMEM 10% FBS. Then, 100 µL per well were distributed in 96-well-conical-plates (non-treated surface) and centrifuged 1 min at 200 × *g*. Spheroids were harvested the next day and transferred in a collagen type I suspension (2 mg/ml) (BD/corning) (354249) supplemented with 1% FBS in the presence of the indicated treatments. After collagen polymerization, DMEM 1% FBS (+/– treatment) were added to the top of each well to prevent any gel desiccation. Invasion was monitored by fully automated *Dmi6000b Wide Field* Fluorescence *Microscope* (Leica Microsystems, Wetzlar, Germany) during 48 h.

### Wound-healing assay

Uniform wounds were obtained into the cell culture by using the ibidi Culture-Insert 2 well (ibidi GmbH Munich, Germany, No. 81176). The culture-Inserts were placed in the individual wells of a 12-well plate. In each reservoir, T47D cancer cells (1 × 10^5^ cells) were plated in 100 µl DMEM 10% FBS. The silicon inserts were removed after the cells had undergone confluence. The gaps created were washed with PBS and each well was filled with 1 ml conditioned media from CAF sg Control or CAF sg MCL-1 (conditioning for 48 h in DMEM 1% FBS). Migration of the cells (wound closure) was monitored by fully automated *Dmi6000b Wide Field* Fluorescence *Microscope* (Leica Microsystems, Wetzlar, Germany).

### Cell proliferation

For cell proliferation, we used live-cell imaging using an IncuCyte FLR imaging system (Essen BioScience). Briefly, T47D-eGFP cancer cells (8 × 10^4^ cells) were plated into 24-well plates containing CAFs sg control or sg MCL-1 (1 × 10^5^ cells plated 2 days before and treated or not with S63845 500 nM for 18 h) in DMEM 1% FBS. Plates were scanned every 24 h for 120 h, scanning 9 fields per well. Results are expressed as percentage of fluorescence intensity relative to T0.

### Statistical analysis

Student’s *t*-tests was used for statistical analysis in experiments with two groups and Two-way analysis of variance (ANOVA) was used for statistical analysis for overall condition effects with GraphPad Prism 5.0 Software. All data are presented as mean ± SEM of at least three independent experiments. The symbols correspond to a *P*-value inferior to *0.05, **0.01, ***0.001, and **** 0.0001.

## Supplementary information

authors agreements

Supplementary data

checklist

Supplementary Figure 1

Supplementary Figure 2

Supplementary Figure 3

Original Data File

## Data Availability

RNAseq data are available on Gene Expression Omnibus under the accession number GSE211012.
