# Supplementary material for: Targeting of MCL-1 in breast cancer-associated fibroblasts reverses their myofibroblastic phenotype and pro-invasive properties
Source: Cell Death Dis. 2022 Sep 14;13(9):787. doi: 10.1038/s41419-022-05214-9 (PMC9474880; doi:10.1038/s41419-022-05214-9)

Return-Path: <chloe.lefebvre3@etu.univ-nantes.fr>

Delivered-To: souaze-f@univ-nantes.fr

Received: from 2001:660:7220:385:193:52:103:60 (proxying for

2a01:cb05:8dbf:7500:38a3:9658:993a:a40)

(SquirrelMail authenticated user e200506r)

by webmail-etu.univ-nantes.fr with HTTP;

Tue, 23 Aug 2022 14:22:26 +0200 (CEST)

Message-ID: <87c40352b25e9e80815d5ed9dcd8e8e9.squirrel@webmail-etu.univ-nantes.fr>

In-Reply-To: <7eaa9bd4-5eed-f85a-0403-e9d9a2e861b2@univ-nantes.fr>

References: <22166125117682@mts-ejp-www8.nature.com.nature.com>

<7eaa9bd4-5eed-f85a-0403-e9d9a2e861b2@univ-nantes.fr>

Date: Tue, 23 Aug 2022 14:22:26 +0200 (CEST)

Subject: Re: Fwd: CDDIS-21-4473RRR Initial Quality Check

From: "Chloe LEFEBVRE" <chloe.lefebvre3@etu.univ-nantes.fr>

To: "Frederique Souaze" <Frederique.Souaze@univ-nantes.fr>

User-Agent: SquirrelMail/1.4.17

MIME-Version: 1.0

Content-Type: text/plain;charset=iso-8859-1

Content-Transfer-Encoding: 8bit

X-Priority: 3 (Normal)

Importance: Normal

I confirm that I agree to add my name to this paper.

Chloé Lefebvre

> Dear Julie and Chloé,

>

> Because of your participation in the work, your name has been added as a

> co-author in the following manuscript:

>

> *Targeting of MCL-1 in breast cancer associated fibroblasts reverses

> their myofibroblastic phenotype and pro-invasive properties ***

>

> Thomas L. Bonneaud ^1,2 , Chloé C. Lefebvre ^1,2 , Lisa Nocquet ^1,2 ,

> Agnes Basseville ^3 , Julie Roul ^1,2,4 , Hugo Weber ^1,2 , Mario

> Campone ^1,2,4 , Philippe P. Juin ^1,2,4 and Frédérique Souazé ^1,2

>

> Could you please confirm by email that you agree with this.

>

> Frédérique


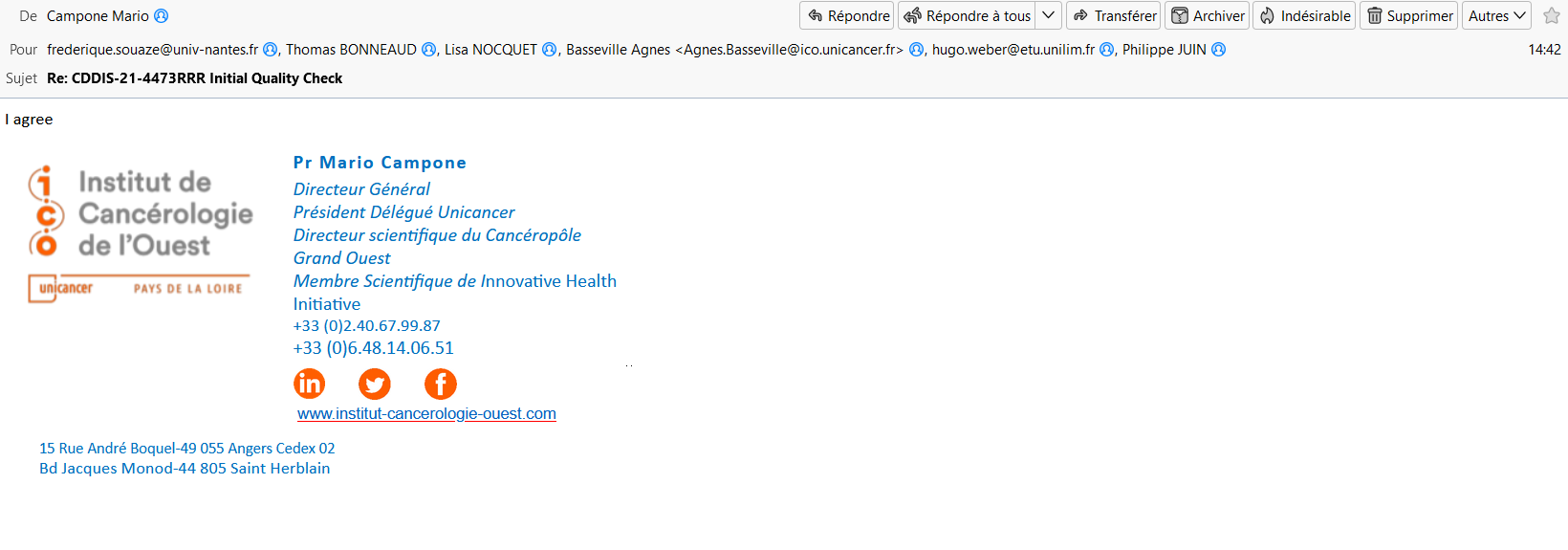


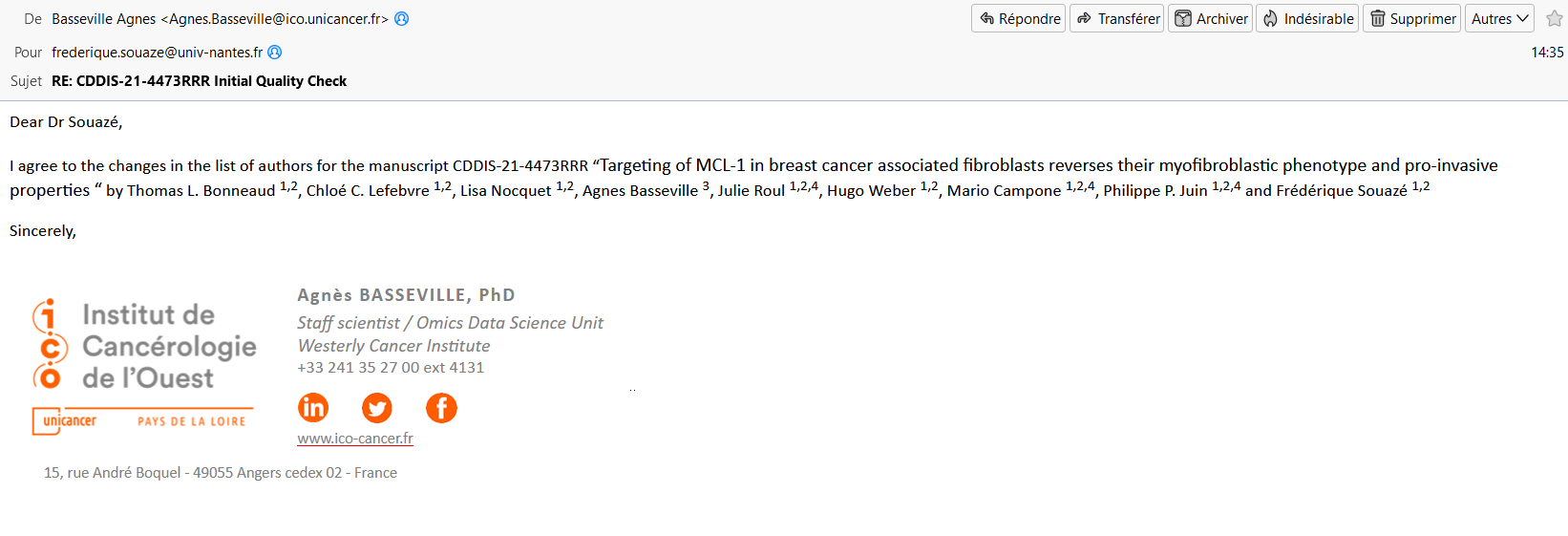


>

Return-Path: <Julie.Roul@univ-nantes.fr>

Delivered-To: souaze-f@univ-nantes.fr

Received: from webmail.univ-nantes.fr (webmail1-lmb.cprv.univ-nantes.prive [172.26.0.60])

by smtp-prv.univ-nantes.fr (Postfix) with ESMTP id 4A0B12EEA3

for <Frederique.Souaze@univ-nantes.fr>; Tue, 23 Aug 2022 14:51:42 +0200 (CEST)

MIME-Version: 1.0

Content-Type: text/plain; charset=UTF-8;

format=flowed

Content-Transfer-Encoding: 8bit

Date: Tue, 23 Aug 2022 14:51:42 +0200

From: Julie ROUL <Julie.Roul@univ-nantes.fr>

To: Frederique Souaze <Frederique.Souaze@univ-nantes.fr>

Subject: Re: Fwd: CDDIS-21-4473RRR Initial Quality Check

Organization: UFR MEDECINE/UMR_S 1307 CRCI2NA

In-Reply-To: <7eaa9bd4-5eed-f85a-0403-e9d9a2e861b2@univ-nantes.fr>

References: <22166125117682@mts-ejp-www8.nature.com.nature.com>

<7eaa9bd4-5eed-f85a-0403-e9d9a2e861b2@univ-nantes.fr>

Message-ID: <8f282f4d07c637aa804576ca1b59cadf@imap.univ-nantes.prive>

X-Sender: Julie.Roul@univ-nantes.fr

User-Agent: Roundcube Webmail/1.1.2

Yes, I agree.

Julie

Julie ROUL

Technicienne de laboratoire

Team 7 "Stress adaptation and tumor escape"

CRCI2NA, UMR 1307 INSERM, UniversitÃ© de Nantes, UniversitÃ© d'Angers

Institut de Recherche en SantÃ©- UniversitÃ© de Nantes

8 Quai Moncousu - BP 70721

44007 Nantes Cedex 1

France

Return-Path: <Thomas.Bonneaud@univ-nantes.fr>

Delivered-To: souaze-f@univ-nantes.fr

Received: from mx1.localdomain (MX1.univ-nantes.fr [193.52.101.135])

by gromel2.univ-nantes.prive (Postfix) with ESMTP id 91E2C1C2FEE0

for <Frederique.Souaze@univ-nantes.fr>; Tue, 23 Aug 2022 14:55:16 +0200 (CEST)

Received: from webmail.univ-nantes.fr (webmail1-lmb.cprv.univ-nantes.prive [172.26.0.60])

by smtp-prv.univ-nantes.fr (Postfix) with ESMTP id 3A0F32725A

for <Frederique.Souaze@univ-nantes.fr>; Tue, 23 Aug 2022 14:55:16 +0200 (CEST)

MIME-Version: 1.0

Content-Type: text/plain; charset=UTF-8;

format=flowed

Content-Transfer-Encoding: 8bit

Date: Tue, 23 Aug 2022 14:55:16 +0200

From: Thomas BONNEAUD <Thomas.Bonneaud@univ-nantes.fr>

To: Frederique Souaze <Frederique.Souaze@univ-nantes.fr>

Subject: Re: Fwd: CDDIS-21-4473RRR Initial Quality Check

Organization: UFR MEDECINE/UMR_S 1307 CRCI2NA

In-Reply-To: <0efc9f22-2698-0590-d59c-5d63bf8e569a@univ-nantes.fr>

References: <22166125117682@mts-ejp-www8.nature.com.nature.com>

<0efc9f22-2698-0590-d59c-5d63bf8e569a@univ-nantes.fr>

X-Priority: 1 (Highest)

Message-ID: <d9238bb8e18eda2c13fe38d6bfa77c3b@imap.univ-nantes.prive>

X-Sender: Thomas.Bonneaud@univ-nantes.fr

User-Agent: Roundcube Webmail/1.1.2

I agree

Le 2022-08-23 14:28, Frederique Souaze a Ã©critÂ :

> Dear Authors,

>

> Following the revision period of the following article.

>

> TARGETING OF MCL-1 IN BREAST CANCER ASSOCIATED FIBROBLASTS REVERSES

> THEIR MYOFIBROBLASTIC PHENOTYPE AND PRO-INVASIVE PROPERTIES Thomas L.

> Bonneaud 1,2, ChloÃ© C. Lefebvre 1,2, Lisa Nocquet 1,2, Agnes

> Basseville 3, Julie Roul 1,2,4, Hugo Weber 1,2, Mario Campone 1,2,4,

> Philippe P. Juin 1,2,4 and FrÃ©dÃ©rique SouazÃ© 1,2

>

> The list of authors has changed, COULD YOU PLEASE CONFIRM BY RETURN

> MAIL YOUR AGREEMENT TO THESE CHANGES.

>

> Sincerely,

>

--

Thomas BONNEAUD

PhD Student

Team 8 - CRCINA (Inserm Nantes)

8 Quai Moncousu

44000 NANTES

Return-Path: <hugo.weber@etu.unilim.fr>

Delivered-To: souaze-f@univ-nantes.fr

Received: from mx1.localdomain (MX1.univ-nantes.fr [193.52.101.135])

by gromel2.univ-nantes.prive (Postfix) with ESMTP id 138671C2FEE0

for <Frederique.Souaze@univ-nantes.fr>; Tue, 23 Aug 2022 15:16:48 +0200 (CEST)

Date: Tue, 23 Aug 2022 13:16:43 +0000

Message-ID:

Hello,

I agree.

Yours sincerely,

Hugo Weber

Return-Path: <Lisa.Nocquet@univ-nantes.fr>

Delivered-To: souaze-f@univ-nantes.fr

Received: from mx1.localdomain (MX1.univ-nantes.fr [193.52.101.135])

by gromel2.univ-nantes.prive (Postfix) with ESMTP id C87491C2FEE0

for <Frederique.Souaze@univ-nantes.fr>; Tue, 23 Aug 2022 17:41:59 +0200 (CEST)

MIME-Version: 1.0

Content-Type: text/plain; charset=UTF-8;

format=flowed

Content-Transfer-Encoding: 8bit

Date: Tue, 23 Aug 2022 17:41:59 +0200

From: Lisa NOCQUET <Lisa.Nocquet@univ-nantes.fr>

To: Frederique Souaze <Frederique.Souaze@univ-nantes.fr>

Subject: Re: Fwd: CDDIS-21-4473RRR Initial Quality Check

Organization: UFR MEDECINE/UMR_S 1307 CRCI2NA

In-Reply-To: <0efc9f22-2698-0590-d59c-5d63bf8e569a@univ-nantes.fr>

References: <22166125117682@mts-ejp-www8.nature.com.nature.com>

<0efc9f22-2698-0590-d59c-5d63bf8e569a@univ-nantes.fr>

X-Priority: 1 (Highest)

Message-ID: <8e17855b9fe193f0ffa8def92f3442d0@imap.univ-nantes.prive>

X-Sender: Lisa.Nocquet@univ-nantes.fr

User-Agent: Roundcube Webmail/1.1.2

Dear FrÃ©dÃ©rique SouazÃ©,

I confirm my agreement to these changes

Sincerely,

Lisa Nocquet

--

Lisa NOCQUET

PhD Student

Team 8 CRCINA, INSERM U1232

IRS UN, 8 quai Moncousu

44007 Nantes cedex 1


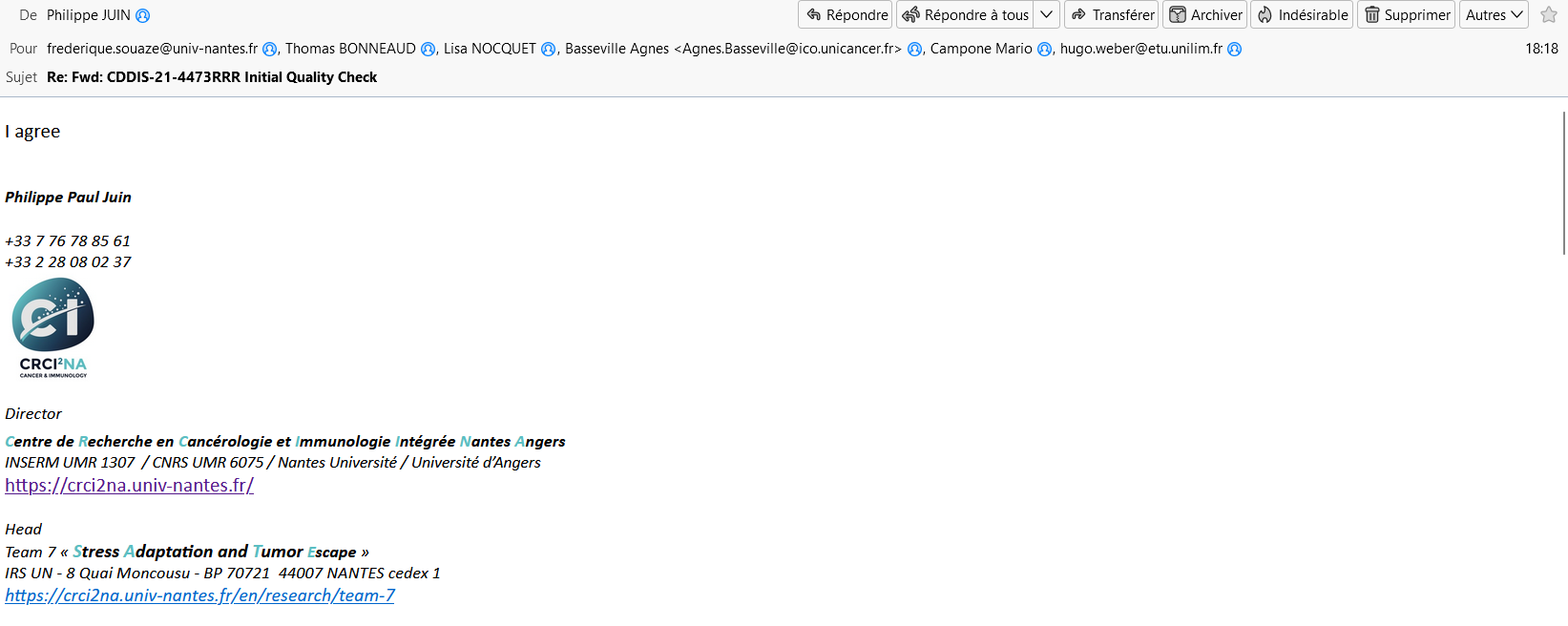

Supplement: Supplementary file 1 — authors agreements [file 41419_2022_5214_MOESM1_ESM.docx]
