# Supplementary material for: Targeting of MCL-1 in breast cancer-associated fibroblasts reverses their myofibroblastic phenotype and pro-invasive properties
Source: Cell Death Dis. 2022 Sep 14;13(9):787. doi: 10.1038/s41419-022-05214-9 (PMC9474880; doi:10.1038/s41419-022-05214-9)
Supplement: Supplementary file 2 — Supplementary data [file 41419_2022_5214_MOESM2_ESM.docx]

**Supplementary Figure 1. A** Multicellular spheroid of T47D cancer cells and three different primary cultures of CAFs were treated or not with S63845 (500nM) for 24 hours in collagen gel (DMEM 1% FBS), T47D cells and CAFs were distinguished by CD90 labelling (as previously described in Louault et al, oncogene 2019), apoptosis was measured by Annexin-V flow cytometry. Data are means ± SEM from three independent experiments. Student t-test, ns: not significant.  **B** Relative Oxygen Consumption Rate (OCR) measured by seahorse in CAFs treated with S63845 (500nM) compared to the control (untreated). Data are means ± SEM from three independent experiments. P value was determined by student t-test. ns: not significant. **C** TOM-20 protein expression levels in primary culture of CAFs treated with S63845 500nM for 18 hours were evaluated using western blots analysis. Actin protein expression was used as loading control. **D** MCL-1 protein expression in CAFs treated by S63845 (500nM) or A1331852 (100nM). **E** Representative mitochondrial colocalization of TOM20 (red) and MCL-1 (green) in CAFs treated or not by S63845 or A1331852

**Supplementary Figure 2. A**. qRT-PCR of ESM1, IL33, HAS2, CCL7 and TMEM158 mRNA in CAFs treated or not by S63845 in presence or not of Mdivi-1. Mean and SEM of three independent experiments are represented as fold change of mRNA level related to untreated. Anova two way, * P<0.1, ** P<0.01, ****P<0.0001.  **B** Volcano plot of the enriched transcription factor. TF: transcription factor. NES, normalized enrichment score. **C** qRT-PCR of ESM1, IL33, HAS2, CCL7 and TMEM158 mRNA in CAFs treated or not by S63845 in presence or not of Mdivi-1. Mean and SEM of three independent experiments are represented as fold change of mRNA level related to untreated. Anova two way, * P<0.1, ** P<0.01, ****P<0.0001. **D** CAFs sg control or silenced for MCL-1 (sg MCL-1) or BAX and BAK (sg BAX-BAK) were treated or not with S63845 (500nM), A1331852 (100nM) or S63845 (500nM) + A1331852 (100nM) for 48 hours in DMEM containing 1% FBS. Top: Protein expression level were evaluated using western blots, Bottom: Quantification of immunofluorescence of TOM-20 (fused or fragmented mitochondria) in different silenced CAFs (as indicated) treated or not with S63845. Randomly generated images set names were randomized for analysis. Around sixty to one hundred cells were analyzed per condition. Counting events were done manually through NIS-Elements software (Nikon software). Fluorescence intensity and positivity staining were determined by positive and negative control comparison, dependent on the experiment. Anova two way, * P<0.1, *** P<0.001,**** P<0.0001, ns: not significant. **E** CAFs sg control or silenced for BAX and BAK (sg BAX-BAK) were treated or not with S63845 (500nM), A1331852 (100nM) or S63845 (500nM) + A1331852 (100nM) for 48 hours in DMEM containing 1% FBS, apoptosis was measured by Annexin-V flow cytometry. Data are means ± SEM from three independent experiments. Two-way ANOVA, ns: not significant.

**Supplementary Figure 3.** Individual values representation of the figure 5F, quantification of Myosin expression and mitochondria fragmentation in CAFs treated by different BH-3 mimetics (as indicated) in presence or not of Mdivi-1.

**Supplementary Figure 4.** Whole un-cropped images of the original Western blots.
