# Supplementary figures and images for: Targeting of MCL-1 in breast cancer-associated fibroblasts reverses their myofibroblastic phenotype and pro-invasive properties
Source: Cell Death Dis. 2022 Sep 14;13(9):787. doi: 10.1038/s41419-022-05214-9 (PMC9474880; doi:10.1038/s41419-022-05214-9)

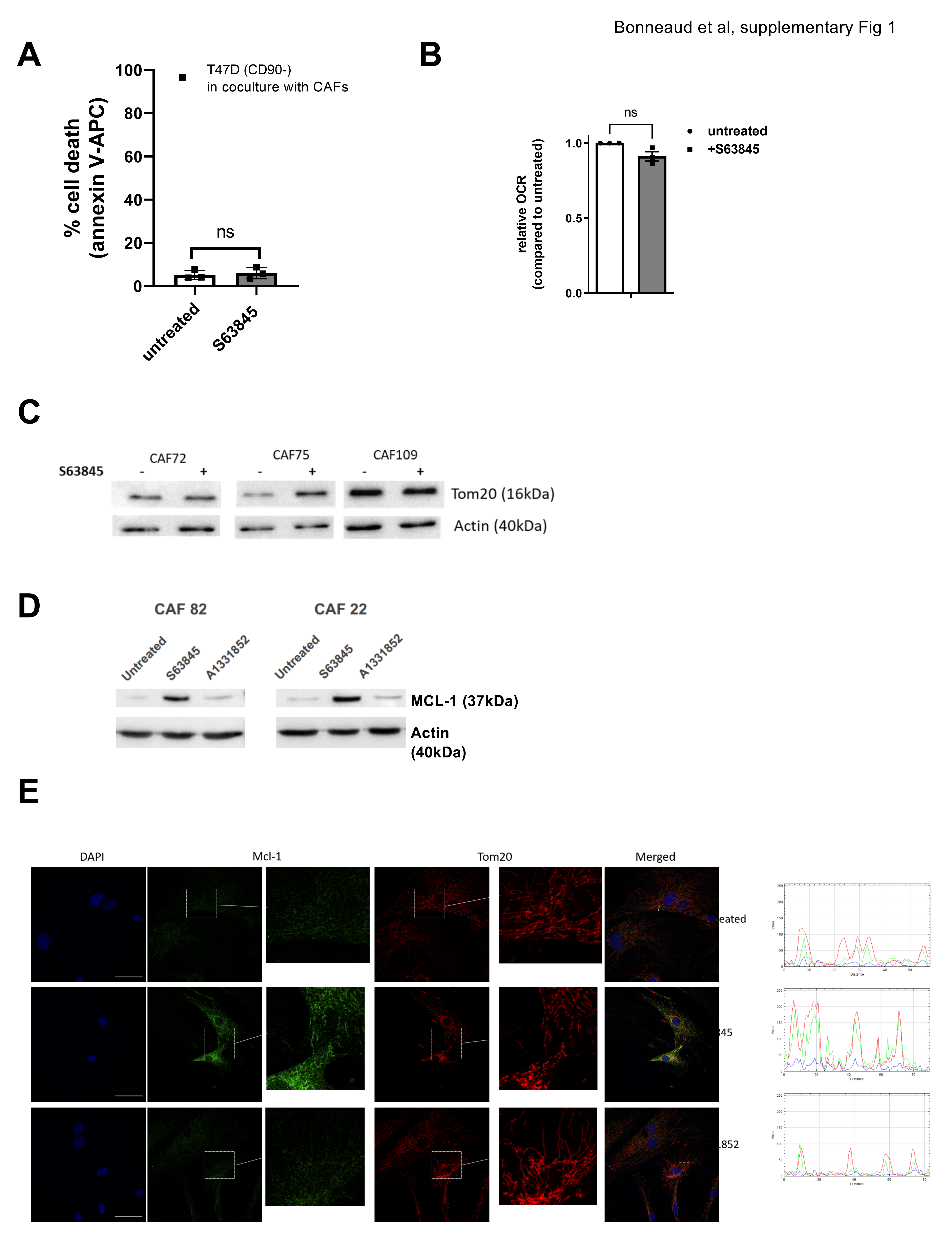

Supplement: Supplementary file 4 — Supplementary Figure 1 [file 41419_2022_5214_MOESM4_ESM.tif]

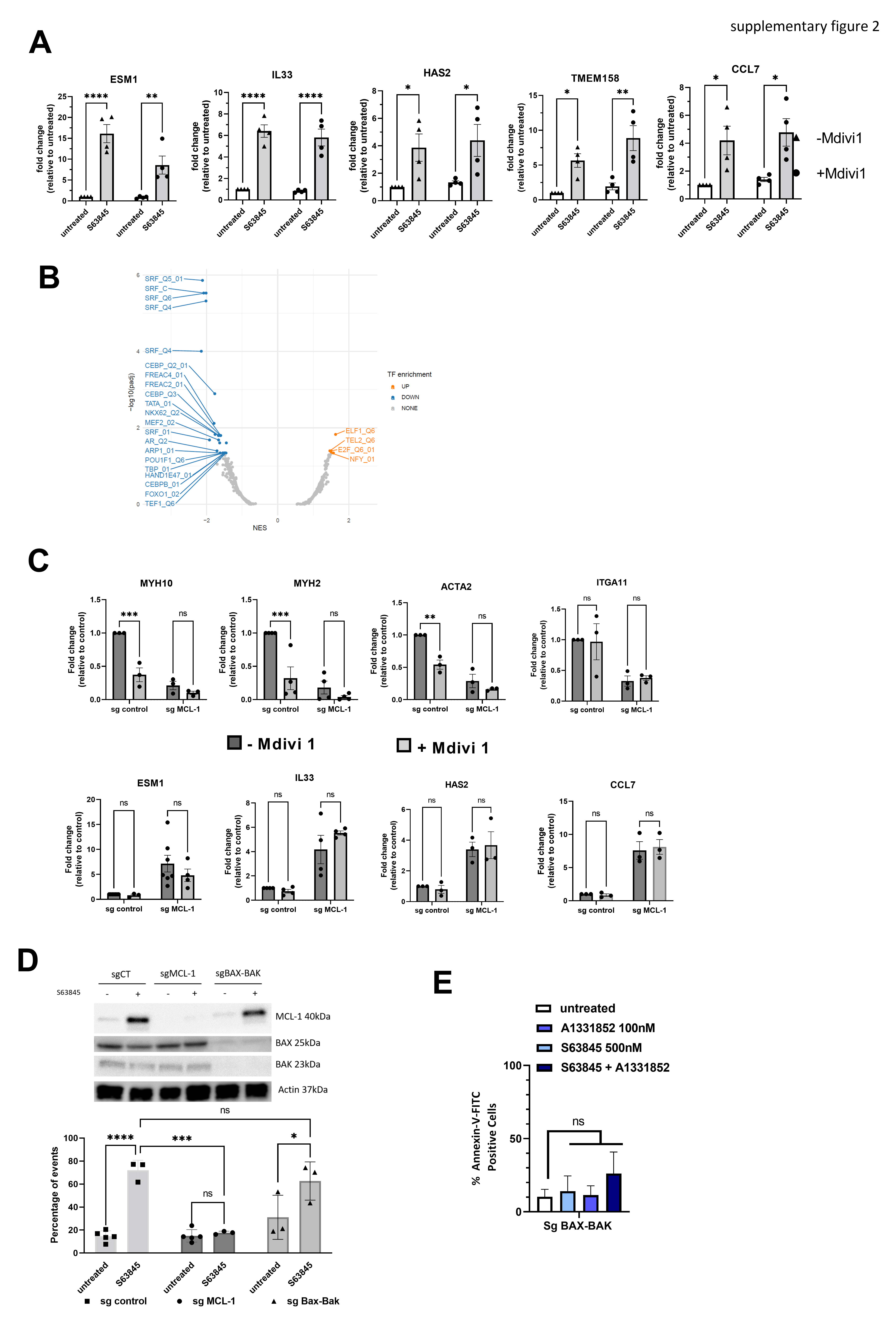

Supplement: Supplementary file 5 — Supplementary Figure 2 [file 41419_2022_5214_MOESM5_ESM.tif]

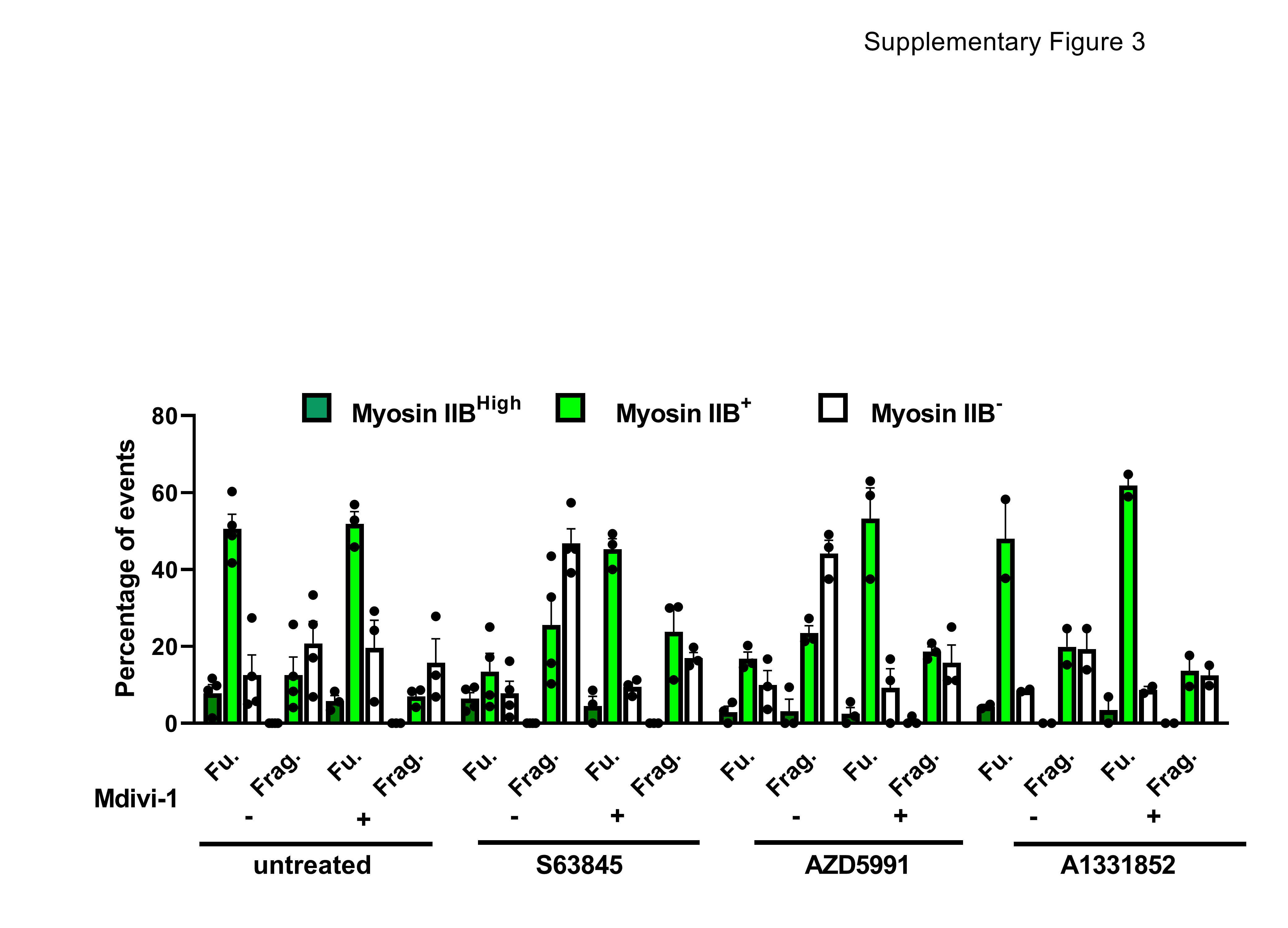

Supplement: Supplementary file 6 — Supplementary Figure 3 [file 41419_2022_5214_MOESM6_ESM.tif]

Figure 5C

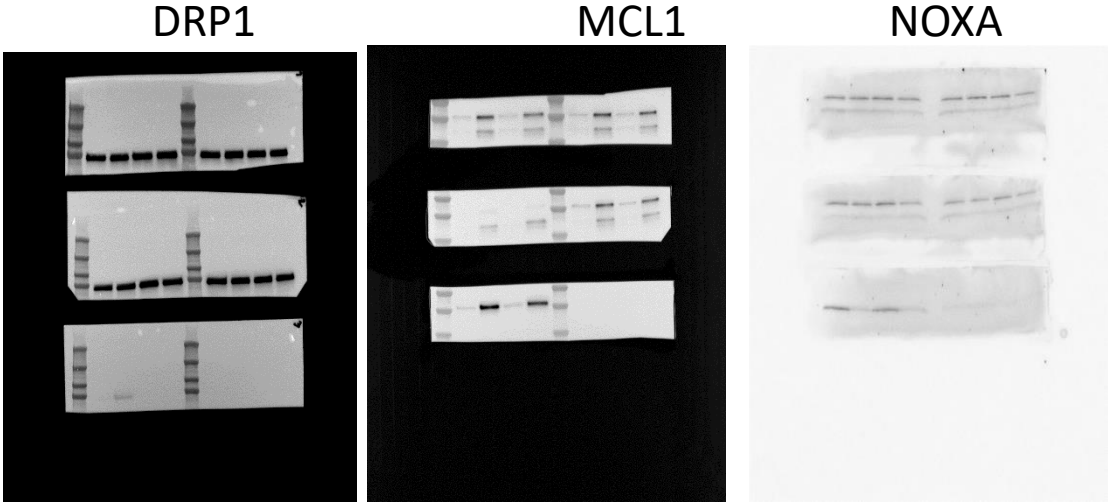

Supp Figure 1C

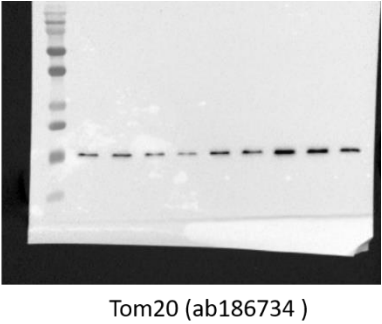

Supp Figure 1D

Mcl-1

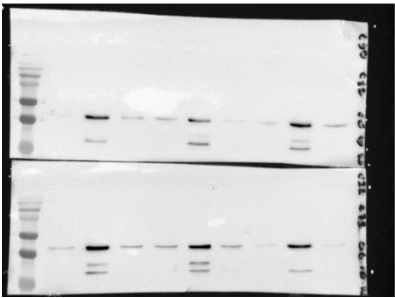

Actin

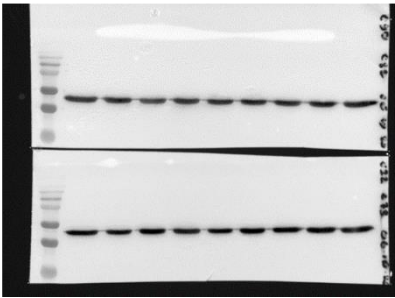

Actin

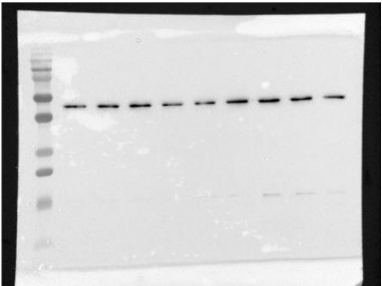

Supp Figure 2D

Bak

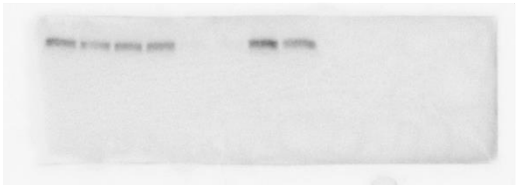

actin

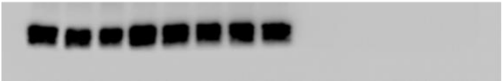

Mcl1

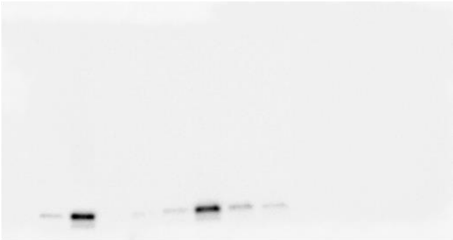

Bax

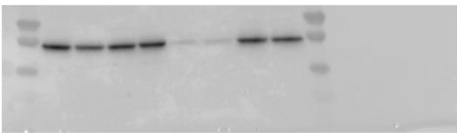

Supplement: Supplementary file 7 — Original Data File [file 41419_2022_5214_MOESM7_ESM.pdf]
